# Official control of plant protection products in Poland: detection of illegal products

**DOI:** 10.1007/s11356-018-1739-2

**Published:** 2018-04-03

**Authors:** Marek Miszczyk, Marlena Płonka, Tomasz Stobiecki, Dorota Kronenbach-Dylong, Kazimierz Waleczek, Roland Weber

**Affiliations:** 1 Pesticide Quality Testing Laboratory, Institute of Plant Protection-National Research Institute Sośnicowice Branch, Gliwicka 29 Street, 44-153 Sosnicowice, Poland; 2 POPs Environmental Consulting, Lindenfirststrasse 23, D-73527 Schwäbisch Gmünd, Germany

**Keywords:** Counterfeit pesticides, Monitoring, Quality of pesticides, Official control, Parallel trade, Poland

## Abstract

Market presence of illegal and counterfeit pesticides is now a global problem. According to data published in 2012 by the European Crop Protection Association (ECPA), illegal products represent over 10% of the global market of plant protection products. Financial benefits are the main reason for the prevalence of this practice. Counterfeit and illegal pesticides may contain substances that may pose a threat to the environment, crops, animals, and humans, inconsistent with the label and registration dossier. In Poland, action against illegal and counterfeit plant protection products is undertaken by the Main Inspectorate of Plant Health and Seed Inspection (PIORiN), the police, the prosecution, and the pesticide producers. Results of chemical analyses carried out by the Institute of Plant Protection - National Research Institute Sośnicowice Branch, Pesticide Quality Testing Laboratory (PQTL IPP-NRI Sosnicowice Branch) indicate that a majority of illegal pesticides in Poland are detected in the group of herbicides. Products from parallel trade tend to have the most irregularities. This article describes the official quality control system of plant protection products in Poland and presents the analytical methods for testing pesticides suspected of adulteration and recent test results.

## Introduction

The production, use, and stockpiling of pesticides have generated extensive pollution globally (Vijgen et al. 2011; Torres et al. 2013; Götz et al. 2013; Toichuev et al. 2017a, b). In the last few decades, pesticide stockpiles have partly been managed under the Stockholm Convention framework, but progress is slow and considerable improvements are needed (Dollimore and Schimpf 2013). Effective management of pesticide stockpiles is possible under the right framework (Vijgen et al. 2013; Weber et al. 2015). Countries such as Moldova, Romania, and Poland have historically managed their unused pesticide stockpiles (Gałuszka et al. 2011; Paun et al. 2014). However, large contaminated sites and stockpiles still exist at pesticide production sites and related landfills (Götz et al. 2013; Vijgen et al. 2011; Weber et al. 2008).

In recent years, the additional challenges of the presence of illegal and counterfeit pesticides on the market have become an increasing global problem (Carter and Durrant 2015; Europol 2011; Karasali et al. 2014; Malkov et al. 2015; Van Hoi et al. 2009). The profitability of the illegal trade in counterfeit pesticides makes it one of the top ten most lucrative organized crime businesses (Europol 2015; Fishel 2015).

The first case of illegal pesticides in Europe occurred in Spain in 2000, when authorities uncovered unregistered pesticides without labels being imported from China (European Commission DG Health and Food Safety 2015). Over the next 5 years, other attempts of introducing illegal pesticides into the market were uncovered in Germany, Italy, the Netherlands, and Poland. According to estimated data published by the European Crop Protection Association (ECPA) in 2012 and included in the document by the European Commission from 2 March 2015 titled “Ad-hoc study on the illegal and counterfeit pesticides in the EU: Executive summary,” illegal and counterfeit pesticides account for over 10% of the worldwide market share of plant protection products. It is estimated that this percent market share exceeds 20–30% in developing countries (Karasali et al. 2014). Based on ECPA data, it is estimated that 8–10% of plant protection products on the European market are counterfeit. Concerning the Polish market, the scale is estimated at 10–15%. There is some evidence suggesting that the amount of illegal pesticides may depend on the geographic location of a European Union (EU) country, being higher for states bordering countries that are not EU members (European Commission DG Health and Food Safety 2015). Some member states may report lower amounts of illegal pesticides due to inadequate control systems in these countries.

The counterfeit pesticide market is growing mainly because of the large profit gained from illegal pesticide sales. The estimated value of global pesticide sales is approximately €44 billion. In the case of Poland, the value of this market is approximately €0.5 billion (European Commission DG Health and Food Safety 2015; Miszczyk et al. 2015a; Miszczyk et al. 2015b). These large amounts of money combined with the legal loopholes and relatively low fines made illegal pesticides trade a fast-growing area of organized crime (Europol 2011). Counterfeit and other illegal pesticides on the market result in losses for the National Treasury and for the chemical manufacturers who produce and sell pesticides within the legal framework, and can also negatively impact farmers’ health and crop yields.

The large risks associated with illegal pesticides need to be emphasized (see below). The composition of registered legal pesticides is precisely determined and monitored by producers. Prior to their registration, which allows for their use and sale on the market, pesticides undergo testing and evaluations designed to assess, among others, the phytotoxicity of their active ingredients, their potential for toxic effects in humans and the environment, and how to use them properly and safely. The evaluation and approval processes take a long time and are very expensive. Counterfeit and other illegal pesticides do not undergo such evaluation and approval processes and thus avoid the associated costs. Furthermore, they are normally produced in countries with weak environmental regulations, making their production cheap. Therefore, illegal pesticides can be sold at a cheaper price resulting in an unfair competition with original products (Miszczyk et al. 2015b).

Due to the lack of testing and production control, counterfeit and other illegal pesticides result in a range of serious risks that can be divided into three different categories:

I. Risk to humans and animals from:

- Increased amounts of residues due to overdosing (inappropriate prescription of procedure)
- Occurrence of unknown and uncontrolled residues in plants, which can be harmful if consumed by people or animals
- Potential for direct adverse effect on sprayer operators
- Additional build-up of pesticide stockpiles

II. Risk to crops from:

- Decreased efficacy or no efficacy at all
- Potential phytotoxic effects of the pesticides or their impurities
- Improper use

III. Risk to the environment from:

- Toxic substances getting into the soil and underground water
- Potentially long-term contamination
- Potential contamination of adjacent crops
- Potential side effects involving beneficial organisms

International and national action is needed to address these risks and to reduce or eliminate counterfeit and other illegal pesticides. This would also benefit the production of safe food for the world’s human population. At the EU level, Europol actively monitors and confiscates illegal pesticides (Europol 2015). The main monitoring and assessment need to be conducted at a national level. However, published studies on monitoring of illegal pesticides in countries are lacking. A study screening for illegal pesticides in Indonesia was published by the International POPs (persistent organic pollutants) Elimination Network IPEN (Dewi and Pertiwi 2006). The research community studied unintentional POPs in pesticides in Australia, and more recently in China (Holt et al. 2010; Huang et al. 2015); these studies however did not specifically address illegal pesticides.

This article describes the activities, approaches, and recent experiences of the Polish authorities regarding the control and assessment of counterfeit and other illegal pesticides, including the practical analysis of pesticides on the market. Such information of practical activities and experiences of national authorities is currently lacking from the international literature and might support developing countries, which often have considerable agricultural activities and pesticide imports but a lack of regulatory framework and monitoring activities, in setting up control measures against counterfeit and other illegal pesticides.

## Materials and methods

### Survey and sampling of pesticides

The study involved samples of chemical plant protection products collected throughout Poland by inspectors from the Main Inspectorate of Plant Health and Seed Inspection (PIORiN). Until 2011, plant protection products were sampled as part of three types of control strategies: scheduled, random, and interventional. Scheduled control visits accounted for 10% of all samples tested each year and involved quality testing of pesticides containing specific active ingredients. Random sampling, which accounted for approximately 73% of all samples tested annually, involved testing product samples collected at points of sale in a random manner. Interventional control, accounting for the remaining 17%, consisted of testing samples suspected of some irregularities, e.g., lack of effective activity; activity different from intended; no documented source (for points of sale); samples detained by the police, border patrol, or collected during interventions.

Due to the very random nature of the control and the weak correlation between the irregularities uncovered in a given year and the schedule of control visits planned for the following year, a new sampling system was introduced in 2012, with two types of official control: basic (targeted) and interventional. The general idea behind the new system is to provide the necessary monitoring at all areas under official control, while also directs particular attention to high-risk areas. High-risk areas are those with the highest number of inconsistent cases discovered in the past, including considerations such as the number of failed certifications issued by the control laboratory that found tested products to be of inadequate quality. Every year, following a data review for the past 2 years, the number of irregularities was divided according to three criteria: the type of permit needed to introduce the product on the market (authorization or parallel trade (PT) permit), product formulation (i.e., the physical form in which plant protection product is marketed, e.g., emulsifiable concentrate, EC; water-dispersible granules; soluble concentrate, SL), and its intended use (herbicide (H), insecticide (I), fungicide (F), other). The review yielded about a dozen different groups (usually 14). A total of 260 samples are collected each year, divided among these groups. Two criteria are used to assign the samples to the groups: the ratio of irregularities to the number of cases in each group, and the volume of sales per group (based on sales data for the past 2 years). Table 1 includes information on the number of groups and their types, as well as the number of pesticide samples per group, scheduled to be sampled in 2014.

**Table 1:** Sample distribution across group (2014)

Group	Type of permit	Intended use	Formulation	Number of samples
1	PT	H	SC	10
2	PT	H	SL	15
3	PT	H	WG	15
4	PT	H	Not (SC, SL, WG)	3
5	PT	F		7
6	PT	Not (F, H)		4
7	Standard	F	EC, FS, WG	25
8	Standard	F	Not (EC, FS, WG)	20
9	Standard	H	SC	28
10	Standard	H	SG, WP	9
11	Standard	H	SL	53
12	Standard	H	WG	16
13	Standard	H	Not (SC, SG, SL, WG, WP)	15
14	Standard	I	EW, WG	10
15	Standard	I	Not (EW, WG)	7
16	Standard	Others	FS, GB, SC, WG	15
17	Standard	Others	Not (FS, GB, SC, WG)	8
Sum				260

The number of samples scheduled to be collected in each of the 16 provinces across Poland was determined based on three criteria: the number of points of sale within a province, product usage based on sales data for the past 2 years, and the crop area within the province. Samples from each group were assigned to provinces randomly using an algorithm developed specifically for that purpose. Because of its large size, the table showing how the samples scheduled for collection were assigned in each province is not included in this article. Each year, the control findings and statistical calculations helped focus the following year’s control efforts on the groups of products most prone to irregularities. Following the introduction of the new system, product samples under the basic control were collected by PIORiN inspectors from the points of sale in a targeted manner, according to specifications developed for each year.

The effectiveness of the new sampling system used during basic control to uncover illegal and fake pesticides was assessed by comparison with the results of lab analyses conducted in 2009–2011. Annually, the Pesticide Quality Testing Laboratory (PQTL) IPP-NRI Sosnicowice Branch tested 300 to 350 samples, including approximately 260 from the official basic control (until 2011, both scheduled and random). For samples of plant protection products covered by the official control, the following were determined: active ingredients, physical and chemical properties, selected impurities, and comparisons with the original product (mainly appearance and analytical profile). Plant protection product formulations are usually a mixture of various chemical substances. Generally, it is not possible to verify the full composition of the formulations and prove compliance with specifications from the registration dossier (Siebers et al. 2014); therefore, comparative methods play an important role in verifying product authenticity.

### Determination of physicochemical parameters and active substances

Pesticide quality tests carried out at the PQTL IPP-NRI Sosnicowice Branch consist of verifying the content of active substances and the basic physical and chemical properties, which were characteristic for a product and documented in the registration process. The following were determined: density, pH, suspension/emulsion/dispersion stability, sieve residue (wet sieve test), wetting time, and foam stability. The tests used methods described in the handbooks of and approved by the Collaborative International Pesticides Analytical Council (CIPAC 2017). For those of active ingredient determinations, the lab used the CIPAC method or manufacturer’s methods included in the registration documents. Where those methods were impossible to use, the lab developed its own internal methods. The manufacturer’s methods and internal methods were validated according to guidelines SANCO/3030/99 rev.4 (European Commission 2000).

### Comparative studies

#### Organoleptic evaluation

Prior to analytical testing, the suspected illegal or counterfeit product sample was subjected to organoleptic evaluation to compare its physical state, appearance, and smell with a reference product and the product registration dossier description.

#### Analytical testing

The PQTL IPP-NRI Sosnicowice Branch developed a number of methods for comparative analysis utilizing different analytical techniques: GC (gas chromatography), HPLC (high performance liquid chromatography), GC-MS (gas chromatography/mass spectrometry), HS-GC-MS (headspace gas chromatography/mass spectrometry), and NIR (near-infrared spectrometry). The applicability of each analytical technique depends on the properties of the substances being analyzed (e.g., polarity, volatility, etc.). The methods were calibrated to match the analytical profile (a chromatogram or spectrum) of the original product (the sample of the original product should ideally be obtained directly from the company holding the original authorization but may also be obtained from the market). The analytical profiles of the original products were then compared to those obtained for the samples of suspected illegal or counterfeit products.

In order to perform comparisons using GC-FID (gas chromatography with flame ionization detector) and HPLC-DAD (high performance liquid chromatography with diode-array detector), two solutions each of the test sample and reference product were prepared. Solution concentration depended on the test material and usually ranged from 0.001–0.01 g/mL. For HPLC, dilution solvents were usually methanol or acetonitrile, and for GC, acetone. A minimum of five injections were performed per each solution. Based on the retention times for each peak of reference products, a range of 3 × SD (standard deviation of retention time for each peak) was set and then the peaks of test samples were checked against the set ranges to see if they matched with the peaks of the original reference products. Additionally, for samples tested using HPLC-DAD, product identity was also determined based on comparisons of UV spectra of peaks of the reference product and test sample. In the case of comparative testing performed using GC-MS and HS-GC-MS, comparisons involved full-scan chromatograms of samples and reference products. Formulation components were identified using mass spectrum libraries (Wiley and NIST, supplied by Agilent, USA). Results were compared to data from product registration dossiers. The identified compounds were compared with the composition of the plant protection product as submitted during authorization, taking into account the results obtained for the original product.

#### Impurities

In selected cases, the contents of the following pesticide impurities were determined:

- 1,2-Dichloroethane, an impurity of ethephon–3 SL formulation products with ethephon content of 480 g/L
- HCB (hexachlorobenzene) and DCB (decachlorobiphenyl), impurities of chlorothalonil–5 SC formulation products with chlorothalonil content of 500 g/L
- Sulfotep, an impurity of chlorpyrifos–39 EC and CS (capsule suspension) formulation products with chlorpyrifos content of 480, 250, and 200 g/L

The above impurities were determined using the in-house methods developed based on the SANCO/3030/99 rev.4 (European Commission 2000) Guidance, using GC-FID, GC-MS, and HS-GC-MS.

## Results and discussions

### The experiences of Polish institutions monitoring illegal pesticides

In Poland, several institutions are involved in activities against illegal pesticides on the market. These include the manufacturers of legal products, the Polish Crop Protection Association (PSOR), the police, customs offices, prosecutor’s office, and the Main Inspectorate of Plant Health and Seed Inspection PIORiN in collaboration with the Institute of Plant Protection - National Research Institute, Sosnicowice Branch (IPP-NRI Sosnicowice). IPP - NRI Sosnicowice has been engaged in quality testing of pesticides since the 1960s, including testing for their places of origin (Stobiecki et al. 2010).

European Parliament and Council (EC) Regulation No. 1107/2009 requires EU member states to conduct independent quality control of pesticides (European Commission 2009). Poland has performed official pesticide quality control for a number of years. Since 2006, this has been implemented under the so-called Multi-year Programme (currently for the years of 2016–2020) by PIORiN and PQTL IPP - NRI Sosnicowice Branch. Polish law requires quality control labs to be Good Laboratory Practice (GLP) certified. The PQTL IPP-NRI Sosnicowice Branch implemented the necessary system in 2008. The main purpose of the official control, the costs of which are covered by the National Treasury, is to uncover any irregularities related to pesticide quality, in particular to look for any products suspected of having been illegally introduced on the market and to verify where they came from. Pesticides were sampled at the points of sale according to guidelines pre-determined by PIORiN inspectors, then forwarded to the lab at IPP-NRI Sosnicowice Branch for testing.

The experience gained by the Pesticide Quality Testing Laboratory (PQTL) of IPP-NRI Sosnicowice Branch allows grouping illegal pesticides into the following categories:

- Pesticides without an active ingredient, or with a different active ingredient
- Pesticides with altered composition, different from what the label says (e.g., diluted)
- Pesticides not registered in a given country
- Pesticides illegally manufactured (from unregistered sources that imitate a legal product, mainly from Asia)
- Pesticides in unoriginal packaging, and/or with an unoriginal label
- Pesticides smuggled in, with a label in a foreign language or no label at all

The above overview shows that mere organoleptic evaluation of the container or its contents can raise suspicions and is a key for monitoring and control of illegal or counterfeit pesticides.

### Results of the assessment

**Fig. 1:**
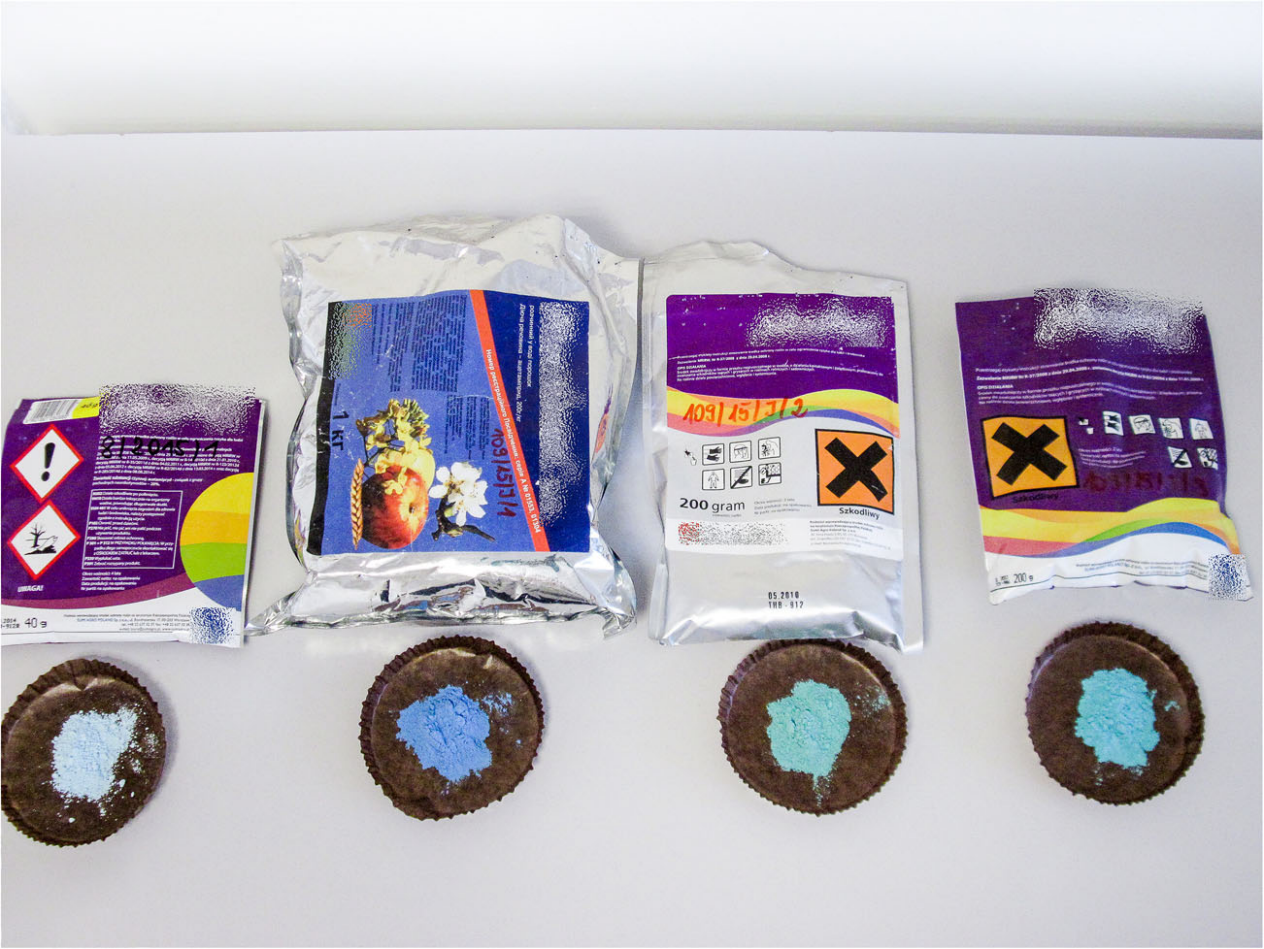
Examples of organoleptic evaluations of a product suspect of being counterfeit against an original product (first from the left)

Plant protection product samples first underwent organoleptic evaluation to verify their state, appearance, color, and smell against the description included in the technical specifications. In most cases, the physical state of the test sample (e.g., solid or liquid) was consistent with the technical specifications. Hence, and due to its subjective nature, this evaluation was considered auxiliary or supplemental. Examples of organoleptic evaluations for color and appearance of a formulation against an original product are shown in Fig. 1.

**Fig. 2:**
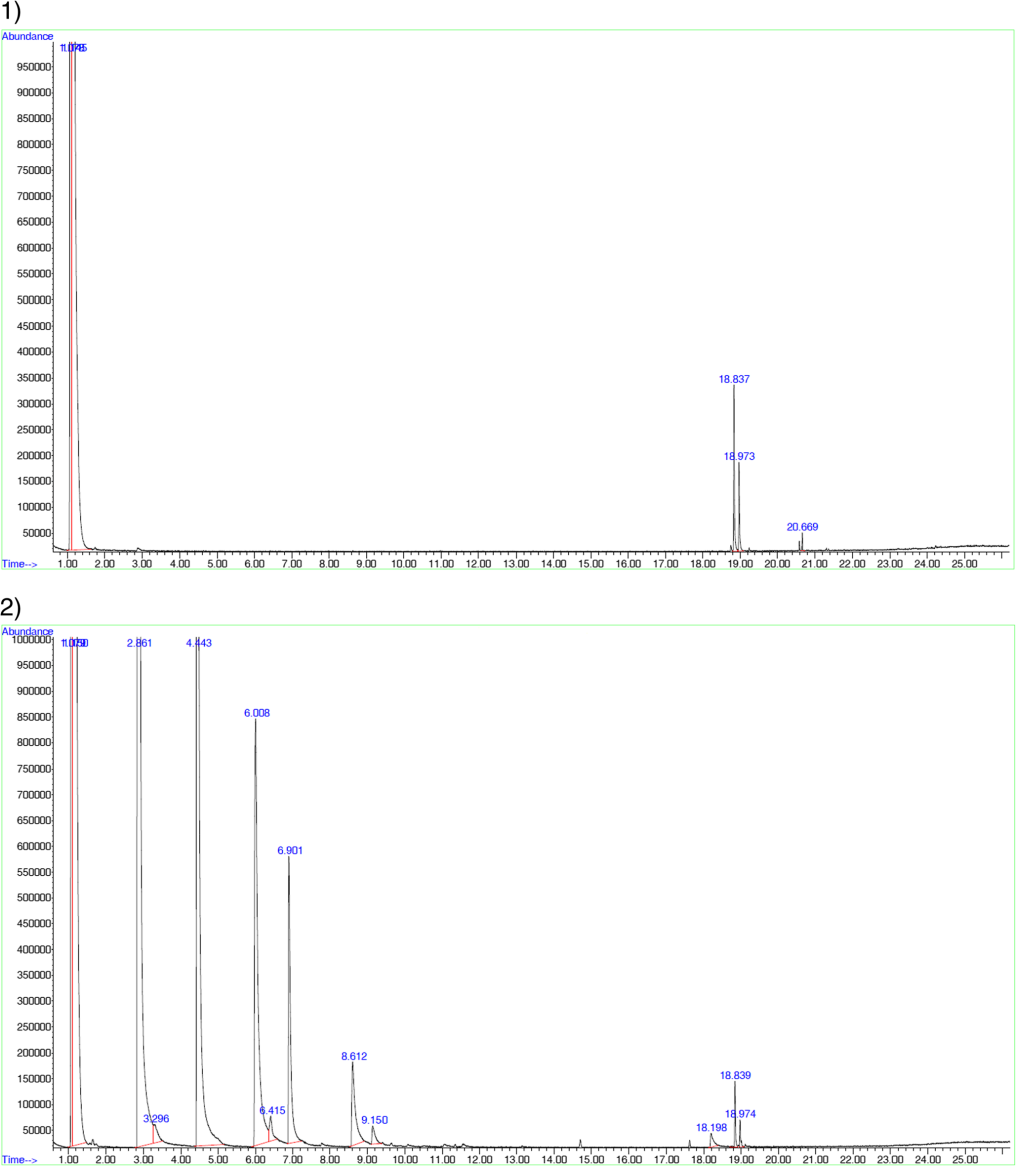
Example of the use of GC-MS technique in comparative studies. **a** Original product. **b** Counterfeit product

Samples suspected of coming from a source other than original were subject of comparative testing using different chromatography techniques. Analytical profiles of test samples were compared with those of the original products. Figure 2 shows samples of chromatograms obtained from the comparative studies.

Tables 2, 3, and 4 include results of official controls for 2009–2011, taking into account the number of uncovered illegal and counterfeit pesticides.

**Table 2:** Number of pesticide samples tested as part of the official control in 2009–2011

Type of control	No. of samples investigated per year		
	2009	2010	2011
Random + scheduled	261	271	268
Interventional	45	78	40
Total	306	349	308

**Table 3:** Number of disqualified products due to inadequate quality (negative certificates) issued for samples tested as part of the official control in 2009–2011

Type of control	No. of negative certificates issued in the following years		
	2009	2010	2011
Random + scheduled	0	2	8
Interventional	37	67	22
Total	37	69	30

**Table 4:** Number of cases of illegal or counterfeit pesticides uncovered during the official control in 2009–2011

Type of control	No. of samples investigated per year		
	2009	2010	2011
Random + scheduled	0	0	6
Interventional	34	53	14
Total	34	53	20

Tables 5, 6, and 7 include results of official controls for 2012–2014 (after introducing the new sampling system), taking into account the number of uncovered illegal and counterfeit pesticides.

**Table 5:** Number of pesticide samples tested as part of the official control in 2012–2014

Type of control	No. of samples investigated per year		
	2012	2013	2014
Basic	275	266	262
Interventional	79	45	48
Total	354	311	310

**Table 6:** Number of disqualified products due to inadequate quality (negative certificates) issued for samples tested as part of the official control in 2012–2014

Type of control	No. of negative certificates issued in the following years		
	2012	2013	2014
Basic	11	6	7
Interventional	40	30	34
Total	51	36	41

**Table 7:** Number of cases of illegal or counterfeit pesticides uncovered during the official control in 2009–2014

Type of control	No. of samples investigated per year		
	2012	2013	2014
Basic	6	4	3
Interventional	30	23	20
Total	36	27	23

A review of test results for 2009–2011 and 2012–2014 indicates a general increase over time in the detection rate of illegal and counterfeit products. In 2009–2011, the number of samples tested annually as part of the scheduled and random control ranged from 261 to 271, and the number of illegal and fake cases uncovered ranged from 0 to 6 (approximately 0.8% of the samples tested). The interventional control involved 40–78 samples tested annually, with 62% fake and illegal products. In 2012–2014, the average percentage of illegal and counterfeit pesticides was 1.6% of the total number of samples tested during basic control, 100% higher than that before the new system was introduced. The complaint-based control carried out in 2012–2014 tested 45–79 samples annually, approximately 42% of which were found to be fake or illegal. The rate of illegal and counterfeit products in relation to the overall number of negative certifications issued was approximately 85% in 2009–2011 and 67% in 2011–2014. This indicates that illegal and counterfeit products are the main types of pesticides disqualified under the official pesticide control.

In 2012–2014, the highest number of counterfeit products was found among herbicides and the lowest among insecticides and other products. In 2012, most irregularities related to illegal sources of pesticides were uncovered among products in parallel trade, which makes it possible to sell pesticides among EU member states as long as a product is properly registered in one of them (in this case, the reference product is a product registered in a given state). The basic reason for disqualifying a product under parallel trade is that it is not considered identical to the reference product, in accordance with Article 52 of Regulation No. 1107/2009. Article 52, section 52.3 says that, “Plant protection products shall be considered as identical to the reference products if: (a) they have been manufactured by the same company or by an associated undertaking or under license in accordance with the same manufacturing process; (b) they are identical in specification and content to the active substances, safeners and synergists, and in the type of formulation; and (c) they are either the same or equivalent in the co-formulants present and the packaging size, material or form, in terms of the potential adverse impact on the safety of the product with regard to human or animal health or the environment” (European Commission Regulation (EC) No. 1107/2009, 2009). Most often, the samples determined as illegal came from the interventional control checks. In 2013–2014, the rate of irregularities detected among products permitted under parallel trade and tested as part of the basic control scheme tended to decrease compared that of previous years.

What makes it more difficult is the fact that the “quality” of counterfeit is getting better and better, and very often the content of active substances and most physical and chemical properties correspond to the specifications of a legal and genuine product. In the majority of cases, determinations of active substances and testing most of the physical and chemical properties are insufficient in order to declare that a product is a fake. A full analysis of a pesticide suspected of coming from an illegal source is practically impossible because it is time consuming and requires a number of expensive testing techniques. This is why the PQTL IPP-NRI Sosnicowice developed a number of methods for comparative analysis utilizing different analytical techniques (GC, HPLC, GC-MS, HS-GC-MS, NIR).

**Fig. 3:**
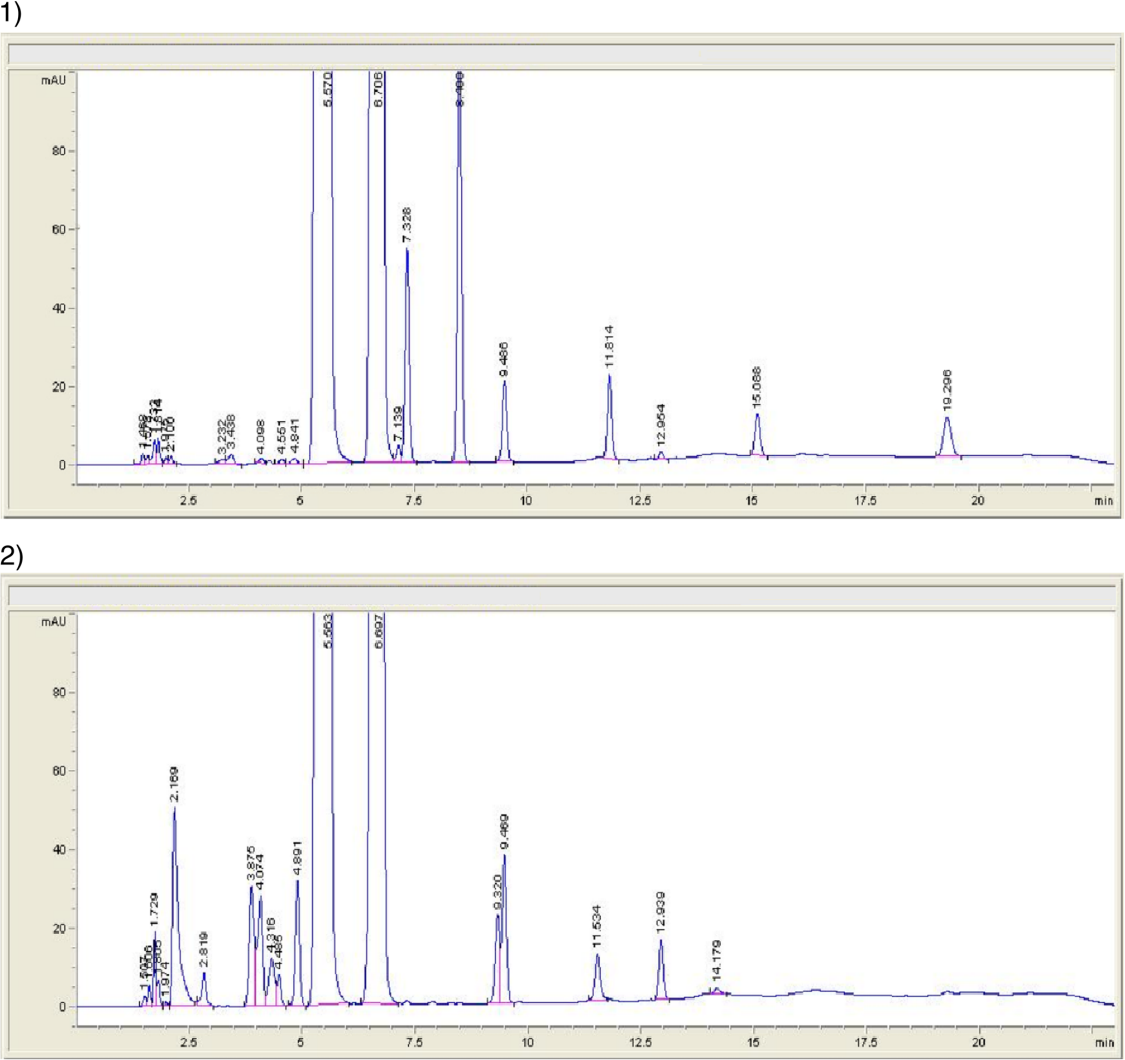
Example of the use of HPLC technique in comparative studies. **a** Original product. **b** Counterfeit product

Examples of the use of comparative methods are shown in Figs. 2 and 3. The figures show the results of the originality tests carried out using GC-MS (Fig. 2) and HPLC (Fig. 3). If the products tested were original, their chromatograms should be practically identical to the chromatograms of the original products. A comparison of the chromatograms shown in Fig. 2a, b indicates that the tested product contains other components than the formulation of the original formulation. This allows the conclusion that the tested product is not identical to the original product. The chromatograms shown in Fig. 3a, b also indicate that the composition of the tested product differs from that of the original product. Peaks with different retention times on the chromatograms of the tested products compared to those on the chromatograms of the original products indicate the presence of substances other than those established in the registration process. The identification of these foreign substances is difficult, and in many cases impossible (especially where it is not possible to use the GC-MS technique). Based on the above results, it can be concluded that both products tested by GC-MS and HPLC are illegal because their composition is different from the one approved in the registration process.

This approach allows for a quick, inexpensive, and effective method of verifying the source of pesticide samples tested by the lab. A fake is usually different from a genuine product in that it contains active substances and other ingredients from unknown sources, obtained through unknown synthesis, therefore contaminated with unknown chemicals, which could be dangerous, e.g., due to their potential toxicity. A counterfeit product may also omit other ingredients like safeners and synergists that affect the proper performance of a product. Comparative testing against original products has many advantages, but a major shortcoming is that it fails to account for differences in batches of the original product. In some cases, this could impact the interpretation of the results and the proper verification of the origin of a given sample. The PQTL IPP-NRI Sosnicowice Branch found a solution to this problem by interpreting the results using chemometric analysis based on physical and chemical data and chromatography results (Miszczyk et al. 2015a).

The maximum allowable impurity content of selected active substances is specified by the Food and Agriculture Organization of the United Nations (FAO) as follows: for HCB and DCB, 0.004 and 0.003%, respectively, of chlorothalonil content in a formulation; for sulfotep, 0.3% of chlorpyrifos content in a formulation; for 1.2-dichloroethane, 0.04% of the declared ethephon content in a formulation (FAO 2000; FAO 2015a; FAO 2015b).

Original products and products found to be counterfeit were tested for selected impurities. Among the three plant protection products containing ethephon, none exceeded the allowable 1.2-dichloroethane limit. Among five chlorothalonil-containing products analyzed as part of this study, none exceeded the allowable concentrations of HCB and DCB. However, among 39 products containing chloropyrifos, one exceeded the allowable sulfotep limit. Comparative studies for this product found that its composition differed from that of the original product.

### Discussion of the experience

The introduction in 2012 of the new system for sampling plant protection products for the purpose of official control conducted in Poland allowed for a combination of targeted control and monitoring of the most problematic areas. A comparison between the inconsistencies detected in pesticide quality by scheduled and random control in 2009–2011 and 2012–2014 indicates that the 2012 approach is more effective. The approach is still a work in progress, trying to adjust to changes in the pesticide market while improving the effectiveness of official pesticide control in Poland. Pesticide quality control, including uncovering of illegal and fake products and testing for impurities and active substances content, is quite a challenge for a control lab because the activities are time, labor, and cost intensive and requires advanced equipment, a variety of analytical techniques, and qualified and experienced personnel able to face ever-increasing challenges. Furthermore, current regulations can be interpreted “broadly,” especially those regarding parallel trade (art. 52), which makes it difficult to impose appropriate sanctions according to Regulation (EC) No. 1107/2009 on companies who release counterfeit and illegal products into the market (European Commission 2009).

The control covered impurities of active substances due to their properties that pose a risk to human health and the environment. HCB and DCB are on the list of persistent organic pollutants attached to the Stockholm Convention, so testing for their presence as impurities in plant protection products, from where they can be released into the environment, is important. (United Nations 2001). Sulfotep is considered a significant impurity of chloropyrifos due to its very high toxicity to aquatic organisms and toxicity to humans through skin contact (MacBean 2012). 1.2-Dichloroethane has potentially carcinogenic effects and needs therefore to be controlled (United States Environmental Protection Agency 2000). Impurities present in a product, even at relatively low levels, can be in some cases very toxic and have a number of adverse effects including delayed neurotoxicity, mutagenesis, or carcinogenicity. Impurities may also enhance toxicity of the product’s active ingredient, may cause phytotoxicity, affect the physical and chemical properties of products, or cause significant bioaccumulation of residues in foods and the environment. Thus, it is necessary to specify the impurity profile for technical materials and technical concentrates of active ingredients. All significant and relevant impurities should be identified, and their maximum allowable limits permitted for technical materials and technical concentrates of active ingredients should be specified in the registration documentation and in FAO specifications (FAO/IAEA 2009). Currently, only few specifications include descriptions of methods recommended for determinations of impurities. Even rarer are the methods that would allow for simultaneous determinations of active substances and their significant impurities in pesticide formulations. Additionally, it should be emphasized that the CIPAC methods used for quality testing of plant protection products, whose availability is one of the basic conditions for creating FAO specifications, are developed by the producers and tend to be highly specific and require the use of dedicated equipment. These methods are developed for a single, concrete active substance, and frequently only for selected formulations in which the substance is available.

Characterizing the impurities for active substances and pesticide formulations is a complicated and lengthy process, designed to ensure the adequate quality of a plant protection product. The reason for this task comes from the fact that the quality of plant protection products is an important factor in their safe use. Testing for impurities of active substances involves their identification and quantification, as well as determination of their biological properties and toxicity.

The introduction of pesticides onto the European Union market is governed by appropriate regulations. According to these regulations, when filing the application to introduce a product onto the market, the product undergoes comprehensive testing and evaluations designed to ensure it is safe to use for crops, people, and the environment. The composition of the product, including its active substances, co-formulants, and any impurities, is specified. In the case of illegal and counterfeit products, this information is unknown. Therefore, even if their impurities are below allowable limits, illegal and counterfeit products cannot be determined as safe for crops, the environment, wildlife, and humans, since they could contain other unknown harmful substances. For instance, significant quantities of methanol in SL formulation and pyridine in SP (water soluble powder) formulation were reported in illegal pesticides. Neither of these compounds is present in the original products. Both methanol and pyridine pose a threat to an operator of sprayer due to their toxic properties (Sapota and Skrzypińska-Gawrysiak 2013; Encyklopedia Techniki, Chemia 1993).

Various campaigns and awareness initiatives directed at food producers and public officials, including police, border patrol agents, and prosecutor’s office personnel, are playing an important role in the fight against illegal and counterfeit pesticides. These national efforts need to be complemented with international activities, such as e.g., monitoring by Europol (Europol 2015), and with better globally harmonized pesticide standards (Handford et al. 2015).

## Conclusion

The effective monitoring, control, and prevention of illegal and counterfeit pesticides on the market is a major task for public authorities, requiring their collaboration with individual farmers, sellers, and legal producers. Recent study results of studies have indicated that counterfeiting of plant protection products is the most serious cause of market failure.

In Poland, an effective system of responsible authorities and governmental laboratories monitoring pesticide quality has been developed since the 1960s. The introduction of the new and more effective system in 2012 for sampling plant protection products for the purpose of official control in Poland allowed tying in the control targeting most problematic areas with monitoring. The approach is still a work in progress, trying to adjust to changes in the pesticide market and improve the effectiveness of the official pesticide control.

The capacity for testing for impurities has been developed over time. Organoleptic evaluation of the container or its contents is a crucial step for monitoring illegal pesticides. Pesticide quality control, in particular testing for impurities and the content of active substances, is a challenge for a control lab because the activities are time, labor, and cost intensive, requiring advanced instruments and analytical techniques, as well as qualified and experienced personnel.

The appropriate regulatory framework for the control of fake pesticides forms the basis for effective control. It is also important to harmonize the regulations in place across the EU member states and at the global level, to ensure consistent interpretation of the laws governing the registration process, introduction on the market, use, and quality control of pesticides.
